# De  Novo Transcriptome Sequencing and Profiling of Ovarian Development of *Argas persicus* Along the Trophogonic Cycle

**DOI:** 10.3390/genes16091107

**Published:** 2025-09-19

**Authors:** Fen Yan, Deyong Duan, Jinzhu Meng, Tianyin Cheng

**Affiliations:** 1 Research Center for Parasites & Vectors (RCPV), College of Veterinary Medicine, Hunan Agricultural University, Changsha 410128, China; yanfen904@126.com (F.Y.);; 2 Guizhou Provincial Key Laboratory for Biodiversity Conservation and Utilization in the Fanjing Mountain Region, Tongren University, Tongren 554300, China

**Keywords:** *Argas persicus*, transcriptome, ovary, differentially expressed genes

## Abstract

**Background**: *Argas persicus* is a hematophagous ectoparasite of poultry and is the vector of several agents infectious to poultry. This study aims to explore the key genes affecting the ovarian development of *A. persicus*. **Methods**: RNA-seq was performed on the ovaries of *A. persicus* before blood-feeding, on the day of engorgement, and 6 days post-engorgement. Utilizing the threshold padj < 0.05 and|log_2_(foldchange)| > 1, differentially expressed genes were identified, and hub genes were determined by constructing protein–protein interaction (PPI) networks. **Results**: A total of 1008 differentially expressed genes were obtained during the feeding period, including 448 up-regulated and 560 down-regulated genes. Further, 2179 differentially expressed genes were screened in the preoviposition stage, including 1957 up-regulated and 222 down-regulated genes. These genes are mainly annotated in functions such as peptidase activity (especially serine protease activity), protein folding, protein assembly, and cell component assembly, and enriched in pathways such as protein processing in endoplasmic reticulum, lysosome, glutathione metabolism, and sphingolipid metabolism. In addition, some proteins that are closely related to ovarian development, including heat shock protein 70, protein disulfide isomerase, paramyosin, troponin I, hexosaminidase, serine protease, Kunitz serine protease inhibitors, and vitellogenin, were obtained. **Conclusions**: These findings fill the gap in the biological data for the ovarian development of soft ticks, provide a reference database for subsequent proteomics research, and offer fundamental support for the screening and development of candidate antigens for anti-tick vaccines.

## 1. Introduction

*Argas persicus* is a member of the order Ixodida, family Argasidae, and genus Argas, and mainly parasitizes domestic fowls and wild birds [1]. Heavy infestation of nymphs can lead to tick paralysis [2]. The life cycle of *A. persicus* includes four stages: eggs, larvae, nymphs (commonly with two to five instars), and adults [3]. During this period, ticks need to blood-feed to grow, molt, and lay eggs. *A. persicus*, when biting infected hosts, can transmit various pathogens, including *Borrelia anserina*, the causative agent of avian spirochetosis; *Francisella tularensis*, responsible for tularemia; *Coxiella burnetii*, which causes Q fever; *Pasteurella avicida*, associated with fowl cholera; and *Acinetobacter haemolyticus* [4,5,6]. Thus, *A. persicus* have become an important vector for the transmission of pathogens, causing significant losses to livestock and poultry breeding.

After the tick feeds, the oocytes grow rapidly and protrude in a grape-like shape. During a certain period in engorged females, oocytes at stages I, II, III, and IV of the Denardi [7] classification can be observed simultaneously in the ovary, but oocytes at stage V are only visible in the oviducts [8]. Studies have shown that in mated female ticks, vitellogenesis was evident 4 days after engorgement, and the number of mature oocytes (stage IV) reaches the maximum 6 days after engorgement [8,9]. After oviposition, the ovaries gradually return to the simple tube-like structure and await the next gonotrophic cycle. Female *A. persicus* usually can undergo 4–6 gonotrophic cycles and can survive for 1 year in a starved state [3,9]. Their characteristics of being multi-host, highly reproductive, and starvation-tolerant increase the harmfulness of the population.

Vaccine immunization is an economical, effective, and environmentally friendly method for tick control. However, the use of vaccines also has limitations. For instance, although the Bm86 subunit vaccine derived from the midgut has been successfully launched on the market, there are regional and tick species differences in its immune efficacy [10,11]. Currently, the functional protein vaccines that had been identified have insufficient protective efficacy and cannot replace the chemical acaricides completely. Therefore, we need to explore more efficient, broader-spectrum, and safer antigens. The development of omics has accelerated the process of functional molecular screening. So far, ovarian transcriptome investigations have been performed in hard ticks, including *Haemaphysalis flava* [12], *Ixodes Ricinus* [13], *Haemaphysalis longicornis* [14], *Amblyomma sculptum* [15], *Rhipicephalus microplus* [16,17], and *Dermacentor albipictus* [18]. However, transcriptome investigations of the ovary of argasid ticks, including *A. persicus*, are unavailable. Therefore, in this study, we used RNA-seq to catalog the transcriptome and to identify potential differentially expressed genes (DEGs) in the ovaries associated with ovarian development at three time points of two key stages (the blood uptake period and the preoviposition period) of the reproductive nutritional cycle in *A. persicus* ticks. Our findings will provide new insights for screening the candidate antigens for anti-tick vaccine development.

## 2. Materials and Methods

### 2.1. Animals and Sample Collection

*A. persicus* were collected from the wall crevices of chicken coops at a farm in Jiayuguan (39°85′N, 98°46′E), China, and were identified and reared at the Parasite and Vector Research Center of Hunan Agricultural University. A total of 420 female ticks, each weighing 19 ± 3 mg and having just completed molting, were allowed to freely mate with twice the number of male ticks under controlled conditions (28 °C, 85% relative humidity). All females originated from the same batch of hatched eggs, and only two nymphal stages were present. After 10 days, 120 female ticks were randomly selected to be dissected to obtain ovaries (OV0). Pools of another 300 were placed on Sanhuang chickens to feed 2 h, and 120 fully engorged ticks (detached freely) were collected to isolate ovaries (OV1), while the ovaries of the 120 females at preoviposition were collected 6 days after blood meal (OV2) and the remaining 60, including unengorged and oviposited females, were discarded. At each time point, the 120 tick ovaries obtained were stored in three cryovials, with each vial containing 40 tick ovaries. This approach ensures that there are three biological replicates for each time point. The ovaries were washed three times with pre-cooled sterile PBS (PH = 7.4) and frozen in liquid nitrogen for RNA-seq and qPCR.

### 2.2. RNA Isolation and Library Construction

Total RNA was isolated from the ovaries in OV0, OV1, and OV2 of *A. persicus* using TRIzol reagent (Invitrogen, Waltham, MA, USA) in accordance with a previously established protocol [19]. The extracted RNAs were detected by Agilent 2100 bioanalyzer (Agilent, Santa Clara, CA, USA), and RNA purity and concentration were evaluated by NanoDrop2000 (Thermo Fisher, Waltham, MA, USA). A total of 3 μg RNA per ovary sample was used to construct each sequencing library using the Ultra™ RNA Library Prep Kit (NEB, Ipswich, MA, USA), according to the manufacturer’s instructions. Briefly, mRNA was enriched from the RNA using Oligo (dT) magnetic beads, and the fragmentation buffer was used to break it into small fragments of about 200 bp. Subsequently, the first and second strands of cDNA were synthesized successively using fragmented mRNA as a template. The purified double-stranded cDNA was subjected to end repair, A-tailing, and ligation of sequencing adapters. The cDNA of approximately 250–300 bp was selected using the AMPure XP system (Beckman Coulter, Brea, CA, USA) for PCR amplification and purification. Finally, the library quality was assessed on the Agilent Bioanalyzer 2100 system (Agilent, Santa Clara, CA, USA) and sequencing was conducted by synthesis on an Illumina NovaSeq 6000 platform (illumina, San Diego, CA, USA) to generate raw reads of 150 bp.

### 2.3. Transcript Splicing and Functional Annotation

To obtain high-quality clean reads, the raw reads were processed to filter the sequences containing adapter, high N rate, length, and low-quality reads using the NGS QC Toolkit (v2.3.3). Additionally, data quality was evaluated using FastQC (http://www.bioinformatics.babraham.ac.uk/projects/fastqc (accessed on 29 March 2022)). The clean reads were assembled into transcripts using Trinity (v2.6.6) [20]. The Corset program (https://code.google.com/p/corset-project/ (accessed on 29 March 2022)) was used to perform hierarchical clustering on the transcripts. In each cluster, the longest transcript was selected as the representative sequence in that cluster and defined as the Unigene. Benchmarking Universal Single-Copy Orthologs (BUSCO, Arthropoda__odb10 database) was used to evaluate the quality of transcript splicing [21]. To obtain comprehensive gene function information, we annotated the Unigenes across seven major databases, including NR (NCBI non-redundant protein sequences), NT (NCBI nucleotide sequences), KEGG (Kyoto Encyclopedia of Genes and Genomes), GO (Gene Ontology), SwissProt (a manually annotated and reviewed protein sequence database), KOG/COG (COG: Clusters of Orthologous Groups of proteins; KOG: euKaryotic Ortholog Groups), and PFAM (Protein family). Subsequently, the Unigene sequence was taken as the reference sequence, and the clean reads of each sample were aligned with the reference sequence using the RSEM (v1.1.17). The number of reads aligned to a certain gene is called the read count.

### 2.4. Differential Expression Analysis of Genes

Considering the impact of sequencing depth and gene length on fragment counts, the Fragments Per Kilobase of exon model per Million mapped fragments (FPKM) is the current method to estimate the gene expression level. Therefore, RSEM was used to convert the read count to FPKM [22]. DEGs between OV1 and OV0, as well as OV2 and OV1, were selected using the threshold padj < 0.05 and| log_2_(foldchange)| > 1 [23]. The expression-based sample clustering and principal component analysis were performed via DESeq2 (1.20.0) [24].

### 2.5. Functional Enrichment Analysis

To obtain the functional information of the genes, we performed the functional annotation on the Unigene sequence. GO [25] and KEGG [26] enrichment analysis was executed by GOseq (1.10.0) and KOBAS (v2.0.12) between OV1 and OV0, as well as OV2 and OV1, respectively. GO terms and KEGG pathways with padj < 0.05 were defined as significantly enriched.

### 2.6. Screening of Candidate Genes Related to Ovarian Development

The DEGs were mapped for coding sequence (CDs) prediction according to the priority order of NR protein library, SwissProt protein library, and TransDecoder (version 3.0.1) software. The CDs were uploaded to the STRING 12.0 database (http://string-db.org/ (accessed on 19 June 2025)) to obtain their predicted protein–protein interaction (PPI). PPI networks with scores greater than 0.7 and interaction relationships less than 10 were retained. Visualization of gene interaction was achieved using Cytoscape (v3.7.1). The hub gene was identified through the Maximum Clique Centrality (MCC) algorithm of its plugin cytoHubba [27], and the genes related to ovarian development were obtained in combination with the literature analysis.

### 2.7. Quantitative Real-Time PCR (qPCR) for mRNA Quantitation

Total RNA (1 μg) remaining after the construction of the ovarian library was used for cDNA synthesis through EasyScript^®^ One-Step gDNA Removal and cDNA Synthesis SuperMix (Transgen, Beijing, China). We selected seven DEGs for validation, including the candidate genes paramyosin, vitellogenin, and heat shock protein 70, as well as additional DEGs: fatty acid-binding protein, chitinase, and ferritin heavy-chain. Following the method described by Kim [28], we determined that the β-actin gene would serve as the reference gene for this experiment. qPCR was performed using a 20 μL reaction volume containing 10 μL of 2 × PerfectStart^®^ Green qPCR SuperMix (Transgen, Beijing, China), 0.8 μL of forward and reverse primers, 4 μL of cDNA, and 5.2 μL RNase and DNase-free water. The qPCR reaction was run on a ABI7300 platform (Applied Biosystems, Foster City, CA, USA) set at 95 °C for 30 s, 35 cycles of 95 °C for 10 s, 60 °C for 30 s, and 72 °C for 10 s. β-Actin and glyceraldehyde 3-phosphate dehydrogenase (GAPDH) gene were used as internal control genes and the relative expression of the genes was calculated using the 2^−△△CT^ method. A significant difference was statistically evaluated with Student’s t-test using SPSS 26.0. The primers used for the differential gene expression analyses are listed in Table 1.

## 3. Results

### 3.1. Quality Analysis of RNA Sequencing

A total of 201,335,043 clean reads were obtained from the nine ovary samples after quality control. The ratios of clean reads/raw reads were all greater than 89.97%. Meanwhile, the GC content ranged from 51.83 to 54.37%. The percentages of Q20 base were all greater than 96.07%, and the percentages of Q30 base were all greater than 90.23%, indicating that the sequencing results were highly reliable. After assembly, 163,186 transcripts (N50 2111 bp) were obtained, and the results of Corset hierarchical clustering were combined to define the Unigene. We obtained 63,500 Unigenes (N50 1693 bp) with lengths ranging from 301 bp to 22,653 bp. In addition, 32,440 annotated genes (51.08%) were obtained and were used in the subsequent analyses (Table 2). After clustering the Trinity database and removing redundancy with the Corset, the BUSCO results indicated an increase in single-copy orthologs and a decrease in duplicate orthologs. The BUSCO completeness score for Unigenes was 91.6%, demonstrating high-quality assemblies suitable for further analysis. (Figure S1, Table S1).

### 3.2. The Profiling of DEGs of A. persicus Ovaries

**Figure 1:**
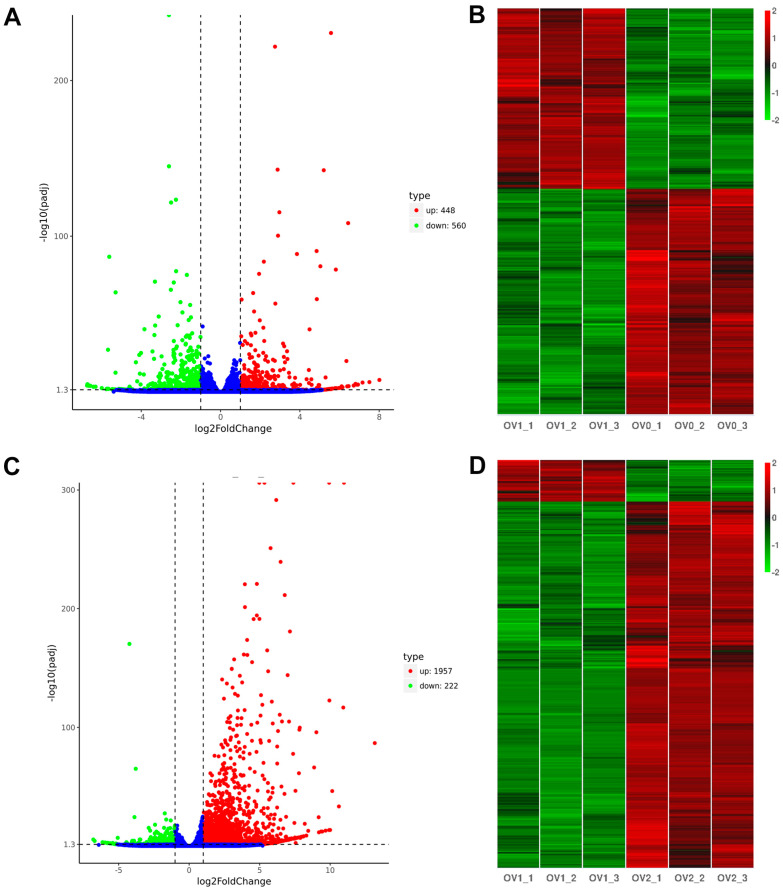
Volcano maps and hierarchical clustering analysis showing the expression profiles of genes between OV1 and OV0 (**A**,**B**), as well as OV2 and OV1 (**C**,**D**), in *A. persicus*. In the volcano plots (**A**,**B**), red dots represent upregulated genes, green dots indicate downregulated genes, and blue dots represent genes with no significant differences. The differentially expressed genes were selected using the threshold padj<0.05 and| log2(foldchange)| > 1.

In total, 1008 DEGs were detected, including 448 up-regulated and 560 down-regulated genes in the OV1 and OV0 ovaries (Figure 1A). Furthermore, we identified 2179 DEGs between the OV2 and OV1 ovaries, containing 1957 up-regulated and 222 down-regulated genes (Figure 1C). Hierarchical clustering showed distinguishable expression patterns of DEGs in the samples (Figure 1B,D). The top 10 up-regulated and down-regulated DEGs were preferentially selected from the blood-sucking and preoviposition periods, respectively. Detailed information can be found in Table 3 and Table 4.

### 3.3. GO Analysis of DEGs

**Figure 2:**
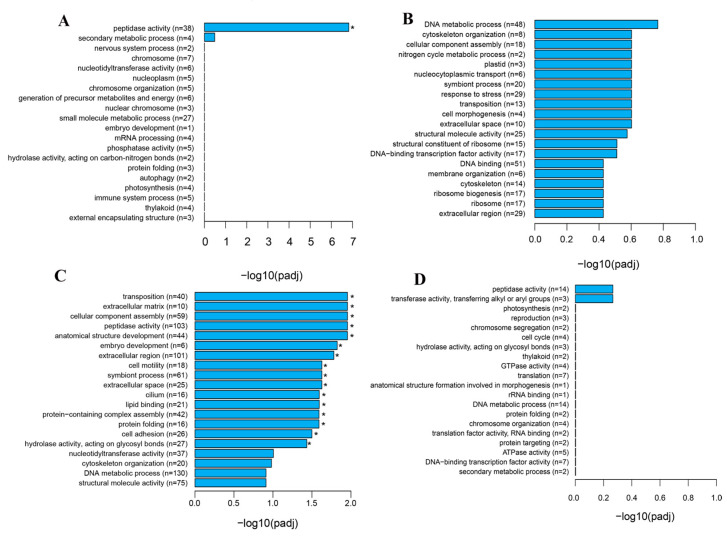
Gene ontology (GO) enriched terms associated with the DEGs in OV1 vs. OV0 (**A**,**B**) and OV2 vs. OV1 (**C**,**D**) of ovaries from *A. persicus.* (**A**,**C**) Up-regulated DEGs, (**B**,**D**) down-regulated DEGs. The top 20 most significant GO terms were illustrated for each compared pair. The mark symbol * indicates significantly enriched GO terms assigned to the differentially expressed transcripts (padj < 0.05). The X-axis represents the −log10 (*p*-value) of the enrichment analysis. The Y-axis indicates the GO terms, with “*n*” in parentheses representing the number of enriched genes.

GO enrichment analyses were performed on the DEGs. The up-regulated genes of OV1 vs. OV0 were enriched in 100 GO terms, among which peptidase activity was significantly enriched, and the down-regulated genes were enriched in 118 GO terms, although none of them were significantly enriched (Figure 2A,B). The up-regulated genes of OV2 vs. OV1 were enriched in 130 GO terms, of which 17 were significantly enriched, including nutrition-related functional categories, such as peptidase activity, hydrolase activity, and acting on glycosyl bonds. Furthermore, up-regulated genes related to peptidase activity can be further annotated to serine-type peptidase activity, metallopeptidase activity, cysteine-type peptidase activity, aspartic-type peptidase activity, and so forth. The down-regulated genes of OV2 vs. OV1 were enriched in 87 GO terms, but none of them were significantly enriched (Figure 2C,D).

### 3.4. KEGG Analysis of DEGs

**Figure 3:**
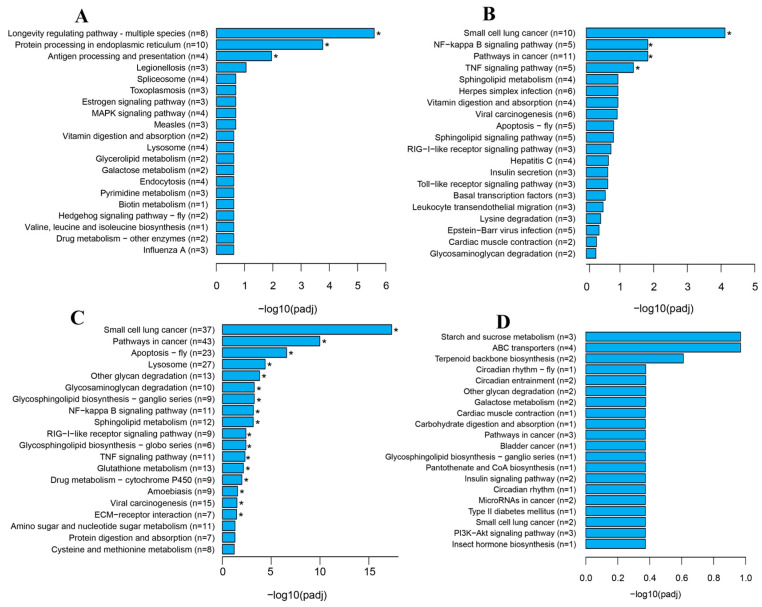
Statistics of the top 20 KEGG pathways for the DEGs in OV1 vs. OV0 (**A**,**B**) and OV2 vs. OV1 (**C**,**D**) of ovaries from *A. persicus*. (**A**,**C**) Up-regulated DEGs, (**B**,**D**) down-regulated DEGs. The top 20 most significant KEGG terms were illustrated for each compared pair. The mark symbol * indicates significantly enriched KEGG terms assigned to the differentially expressed transcripts (padj < 0.05). The X-axis represents the −log10 (*p*-value) of the enrichment analysis. The Y-axis indicates the KEGG pathway, with “n” in parentheses representing the number of enriched genes.

The DEGs were also annotated using KEGG to identify the enriched pathways. The up-regulated genes of OV1 vs. OV0 were enriched in 88 pathways, of which 3 pathways were significantly enriched, including protein processing in endoplasmic reticulum and antigen processing and presentation (Figure 3A). The down-regulated genes of OV1 vs. OV0 were enriched in 156 pathways, 4 of which were significantly enriched, including NF-kappa B signaling pathway and TNF signaling pathway. Furthermore, the up-regulated genes of OV2 vs. OV1 were enriched in 240 pathways, of which 17 pathways were significantly enriched, including lysosome, NF-kB signaling pathway, other glycan degradation, sphingolipid metabolism, RIG-I-like receptor signaling pathway, glutathione metabolism, and ECM–receptor interaction (Figure 3C). The down-regulated genes of OV2 vs. OV1 were enriched in 73 pathways, but none of them were significantly enriched (Figure 3D).

### 3.5. Candidate Genes Related to Ovarian Development

**Figure 4:**
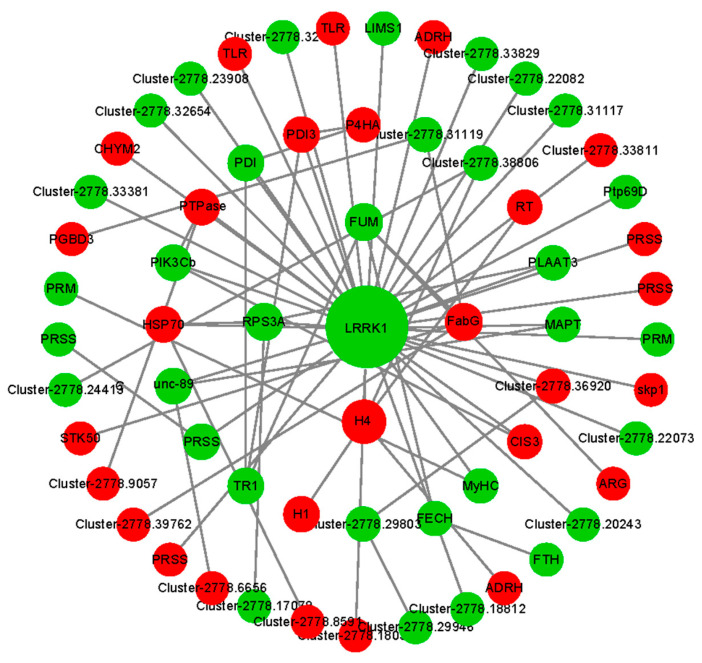
Potentially functional genes compose the interactive network between OV1 and OV0 ovaries. The red and green represent up-regulation and down-regulation, respectively. Larger node sizes indicate higher degree values.

**Figure 5:**
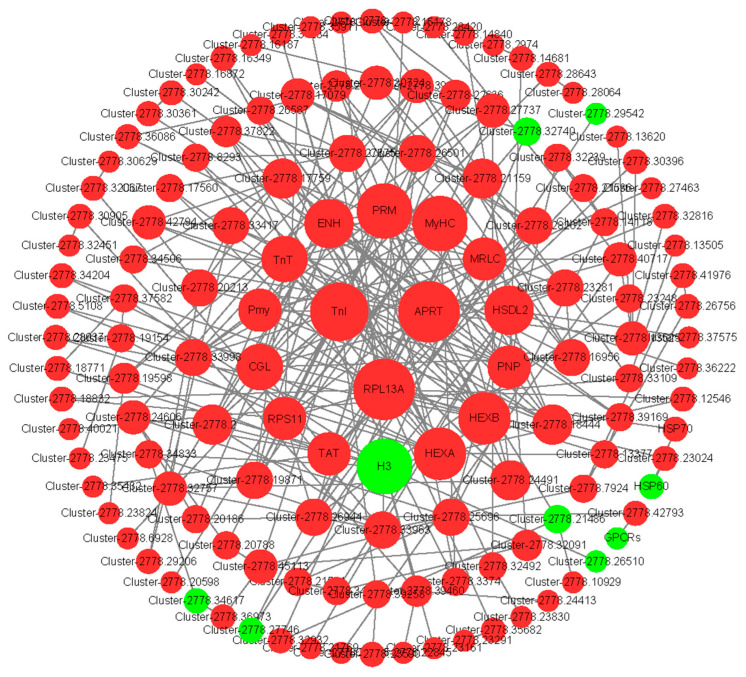
Potentially functional genes compose the interactive network between OV2 and OV1 ovaries. The red and green represent up-regulation and down-regulation, respectively. Larger node sizes indicate higher degree values.

To further explore the functional relationship between DEGs, a PPI regulated network was constructed (Figure 4 and Figure 5). During the blood-feeding and preoviposition periods, 316 and 814 differentially expressed gene-encoded CDs, respectively, matched the protein sequences of *Ixodes scapularis* (NCBI Taxonomy ID: 6945). In the network, there were 264 nodes and 68 interaction relationships during the feeding period (*p* < 5.27 × 10^−5^), while there were 197 nodes and 398 interaction relationships during the preoviposition period (*p* < 1.0 × 10^−16^). Ten hub genes were identified using the MCC algorithm of the cytoHubba plugin (Table 5 and Table 6). The hub gene coding sequences were compared with homologous proteins from the Ixodes scapularis (NCBI taxonomy ID: 6945) and UniprotKB database, with the results presented in Table 5 and Table 6. During the blood-feeding stage, fumarase (FUM) is related to energy metabolism, protein disulfide isomerase (PDI), protein disulfide isomerase 3 (PDI3), and 40S ribosomal protein S3a (RPS3A) are related to protein synthesis and modification in the blood-feeding phase. 3-oxoacyl-(acyl carrier protein) reductase (FabG) is a key enzyme for fatty acid synthesis (Table 5). Heat shock protein 70 (HSP70) is a molecular chaperone, PIK3Cb and PTEN jointly regulate the PI3K/AKT/mTOR pathway, and H4 is involved in the regulation of the cell cycle (Table 5). Among these, seven hub-gene-encoded proteins demonstrate over 71% amino acid sequence similarity with proteins from other tick species, as indicated in Table S2. During the preoviposition stage, sex-hormone-related enzymes such as hydroxysteroid dehydrogenase protein 2 (HSDL2), cytoskeletal-protein-related proteins troponin I (TnI), paramyosin (PRM), and myosin heavy chain (MyHC), as well as hydrolases like adenine phosphoribosyltransferase (APRT), beta-hexosaminidase subunit beta (HEXB), and beta-hexosaminidase subunit alpha (HEXA), were included (Table 6). Notably, six hub-gene-encoded proteins demonstrate over 80% amino acid sequence similarity with proteins from other tick species, as shown in Table S3.

### 3.6. Verification of DEGs

**Figure 6:**
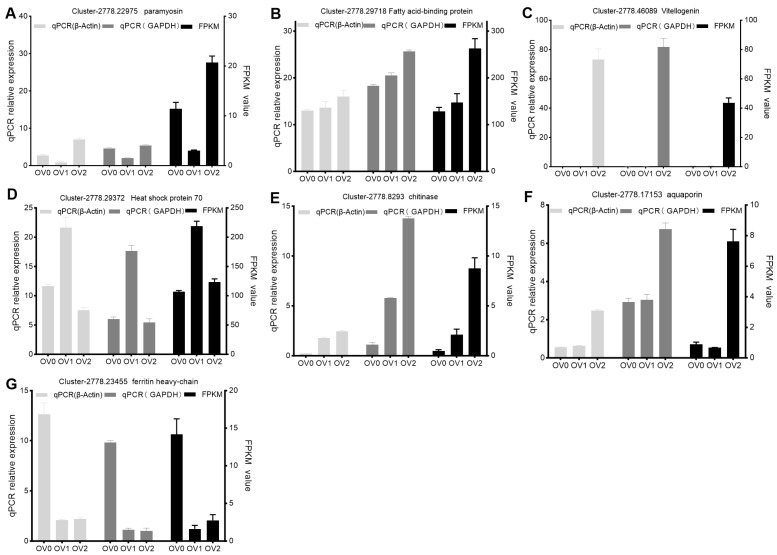
Verification of the expression of DEGs at the three time points of two key stages in *A. persicus*. (**A**–**G**) presents the qPCR (**left**: β-Actin as internal control; **middle**: GAPDH as internal control) and RNA-seq (**right**) analyses for the selected seven genes: paramyosin, fatty acid-binding protein, vitellogenin, heat shock protein 70, chitinase, aquaporin, and ferritin heavy-chain. β-Actin (**left**) and GAPDH (**middle**) were utilized as internal reference genes. The left Y-axis indicates the relative gene expression levels derived from the qRT-PCR analysis, while the right Y-axis displays the FPKM values obtained from RNA sequencing. The X-axis represents ovary samples at different time points. The error bars indicate the standard deviation of three biological replicates.

To verify the results of RNA-seq data, seven DEGs were selected for qPCR analyses. qPCR data (Figure 6) suggest that the RNA-seq results are reliable and can represent the virtual expression pattern at the three time points of two key stages (the feeding period and the preoviposition period) of the reproductive nutritional cycle in *A. persicus*.

## 4. Discussion

During blood-feeding, host immunoglobulins enter the tick’s midgut along with the blood meal, traverse the intestinal wall in their original form, and subsequently enter the hemolymph, where they can reach the ovaries and bind to specific antigens [29,30]. This binding within the tick’s ovaries suggests that targeting ovarian proteins as antigens presents a strategic approach for the long-term and sustainable control of tick populations by regulating their reproduction. Research indicates that the ovarian protein vitellin [31] provides partial protection to sheep against *R. microplus*, while the recombinant protein vaccine of follistatin-related protein [32] significantly reduces tick egg production. Furthermore, the knockdown of the vitellogenin receptor not only reduces fecundity but also disrupts the pathway for *Babesia* spp. to colonize the ovaries, thereby affecting the transovarial transmission of the tick-borne pathogen [33]. This indicates that vaccines developed based on ovarian proteins have considerable potential in preventing tick-borne pathogen transmission. Research suggests that, in addition to protein conservation being a critical factor influencing vaccine efficacy, combined immunization targeting different organs/tissues of ticks may enhance IgG responses [34,35]. Therefore, constructing a tick ovarian transcriptome library and screening for candidate antigens is essential for the development of anti-tick vaccines.

*A. persicus* has a very short feeding time, and mostly feeding takes place within 20–30 min, so these ticks were fully engorged within two hours. During this period, no changes in morphology were observed; however, significant differential expression was noted between ovarian transcripts (OV1 vs. OV0) [36]. The up-regulated genes are mainly enriched in peptidase activity and involved in the protein processing in endoplasmic reticulum pathway, indicating that blood uptake behavior can rapidly stimulate the ovarian transcription of peptidase activity and genes related to protein synthesis. The antigen processing and presentation (up-regulation), NF-kappa B signaling pathway (down-regulation), and TNF signaling pathway (down-regulation) related pathways are associated with immunity and stress [37,38,39]. Previous studies [37,38,39] mostly focused on the relationship between ticks and tick-borne pathogens, and the molecular mechanism that promotes ovarian development remains unclear. After engorged with blood, the *A. persicus* will detach from the host. Subsequently, the oocytes protruding from the ovaries grow rapidly for 5 to 6 days until they mature (OV2 vs. OV1) [36]. During this period, the number of up-regulated genes was significantly greater than that of down-regulated genes. The up-regulated genes were significantly enriched in the lysosome pathway, glutathione metabolism, and sphingolipid metabolism, which all relate to the meiosis of germ cells [40,41,42,43,44,45] and may be involved in cell proliferation. Ticks require a large amount of energy during physiological processes such as yolk formation, egg-laying, and embryonic development [40,46]. Research indicates that peptidase is present in the highest concentrations during the final stage of vitellogenesis, where it hydrolyzes proteins to release energy [40,46]. In this study, the up-regulated genes annotated to peptidase activity and glycosylated bond hydrolases suggest that genes related to nutritional digestion are indispensable during ovarian development.

We observed that among the top 10 up-regulated genes during the blood-feeding period (Table 3), serine protease had the largest up-regulation and down-regulation amplitudes from before blood uptake to before oviposition, while its inhibitor was significantly up-regulated in the preoviposition stage (Table 4). Unlike salivary glands [47], the transcripts of serine-type peptidases are the most abundant among the peptidases in the DEGs of the ovary, indicating that serine proteases play a crucial role in this biological process. As a proteolytic enzyme, serine protease is involved in physiological activities such as digestion, development, signal transduction, and immunity [48]. The Nudel gene encoding serine protease in Drosophila can cause female infertility [49] or prevent sperm from entering the egg by participating in the polar development of the egg and influencing the early cleavage process [50,51]. Mutations of the ovarian serine protease gene can cause reproduction to be hindered in pests such as *Plutella xylostella* [52], *Spodoptera litura* [53], *Bombyx mori* [54], and *Ostrinia furnacalis* [51]. In the study of ticks, the vaccine prepared prepared from longistatin, a serine protease, was approximately 73% effective in inhibiting the infestation of *H. longicornis* [55]. Furthermore, tick serine protease inhibitors such as Kunitz-type protease inhibitors [56,57] and serpin [58] play a significant role in regulating tick physiology by inhibiting serine protease activity. Kunitz-type serine protease inhibitors, including IrSPI [59], Doenitin-1 [60], and BmTI-A [56], are mainly involved in the inflammatory and hemostatic processes, which may be the candidate antigens for controlling ticks and developing new antithrombotic drugs in the future. Before egg-laying in this study, the expression level of the Vg (Cluster-2778.46089) was significantly up-regulated (Table 4), which might be related to the massive synthesis of Vg protein in the ovary. After Vg is ingested/synthesized, it is processed into vitellin (Vn) in the oocyte for storage. The content of Vn accounts for more than 85% of the total protein in the oocyte, thereby fulfilling the nutritional requirements during embryonic development [61]. Vg is not only the main nutrient for egg reserve, but also can bind to heme, reduce heme-induced cellular oxidative damage, and even affect the infection and transmission of tick-borne pathogens [62,63].

Three key genes affecting ovarian development during the uptake period, namely, HSP70, PDI, and PDI3, were identified from PPI analysis. HSP70 is widely distributed in various tick tissues; besides its molecular chaperone effect, it also plays an anticoagulant role during the blood-sucking process [64]. Further research has indicated that HSP70 is closely related to the expression of vitellogenin (Vg) and can regulate ovarian maturation [65,66]. The PDI and PDI3 genes discovered in this study belong to the PDI family, which is involved in the folding, assembly, and post-translational modification of proteins in the endoplasmic reticulum and is involved in collagen synthesis [67]. PDI is essential for the embryonic development of *Caenorhabditis elegans* [68]. In addition, specific PDI has the effect of inhibiting the colonization and transmission of tick-borne pathogens [69]. Furthermore, three additional key genes (PRM, TnI, and HEX) affecting ovarian development prior to oviposition, were also determined through PPI analysis. PRM is a muscle protein [70] that exists exclusively in invertebrates and has been utilized to develop vaccines against *Trichinella spiralis* [71], *Schistosoma japonicum* [72], *tapeworm* [73], and poultry red mite [74]. The recombinant protein of PRM and PRM epitope could produce 60.37% and 70.86% efficacy against female *H. longicornis*, respectively [75]. TnI, an essential component of muscle tissue, also plays a role in inhibiting angiogenesis [76]. Immunization of the host with TnI recombinant protein significantly reduced the engorgement rates of larval and adult ticks as well as the female fecundity of *H. longicornis* [77]. HEX, a lysosomal hydrolase comprising two subtypes (HEXA and HEXB) [78], participates in chitin degradation [79] and fertilization processes [78], making it a potential target for insect control. The application of *R. microplus* HEX polyclonal antibody has been shown to decrease the oviposition of *R. microplus* by 26% [80]. Due to the limited availability of genomic library data for soft ticks, we opted to screen hub genes based on the protein–protein interaction (PPI) network constructed from the I. scapularis database. Tables S2 and S3 demonstrate that the amino acid sequences of the proteins encoded by HSP70, PDI, PRM, TnI, and HEXB exhibit over 75% similarity with their homologous proteins in Ixodes scapularis, thereby validating the accuracy of the annotation of these core genes within the PPI network. Furthermore, the coding sequences (CDS) of these five genes show more than 83.4% similarity with homologous proteins from other tick species in the UniprotKB database, indicating the presence of partially conserved sequences. The development of a broad-spectrum anti-tick vaccine is contingent upon the identification of highly conserved tick proteins. The results indicate that HSP70, PDI, PDI3, PRM, TnI, and HEX have the potential to develop anti-tick vaccine antigens.

## 5. Conclusions

This study successfully assembled and constructed an ovarian transcriptome database for *A. persicus* de novo. Analysis of the transcriptome libraries at three time points during the trophogonic cycle revealed functions such as peptidase activity (particularly serine protease activity), protein folding, protein assembly, and cellular component assembly. Furthermore, pathways related to protein processing in the endoplasmic reticulum, lysosomes, glutathione metabolism, and sphingolipid metabolism are crucial for ovarian development. The identified candidates HSP70, PDI, PRM, TnI, HEX, serine protease, Kunitz, and Vg are potential antigens for the development of anti-tick vaccines. Given that some of the DEG-encoded proteins in this PPI network are not involved, and the number of interactions is relatively small, it can be inferred that the identification of PPIs affecting the ovarian development of *A. persicus* is not yet exhaustive. Therefore, it is essential to continue studying the interactions between proteins that influence the ovarian development of *A. persicus*. Additionally, functional validation and immunogenicity testing can be employed to more thoroughly assess the potential of these candidate genes for the development of anti-tick vaccines.

## Figures and Tables

**Table 1:** Primer sequence for qRT-PCR.

Gene ID	Gene	Nucleotide Sequence (5′–3′)	Size (bp)
Cluster-2778.22975	paramyosin	F: CAACCGCCGCATTCACGAGTAC	123
		R: CGTTGGCACTTGGCTTCCTCCT	
Cluster-2778.29718	Fatty acid-binding protein	F: TAGGATGGGGCGGAAATGTG	125
		R: CTTCATCGTCCACTGGTCCC	
Cluster-2778.46089	Vitellogenin	F: TGGCAGCAACTCCTCCGTCAAC	194
		R: TGGTCAGCACAAGTGGCGACAA	
Cluster-2778.29372	Heat shock protein 70	F: CGACATGGACGCCAACGGTATC	139
		R: CTGTTCAGCCTCCTTCAGCATCC	
Cluster-2778.8293	chitinase	F: GCTCTGAAATCGAGCGCAAG	138
		R: CAGACAGCGAAGTGTTTGCC	
Cluster-2778.17153	aquaporin	F: CGCCCTGGTGTACCTCATTT	106
		R: GGGGTACGTAGCGAAGATGG	
Cluster-2778.23455	ferritin heavy-chain	F: AGACCCTGGATGGAGATGACT	97
		R: CAAGGTCAACCACAGAAGAGC	
Reference 1	β-Actin	F: AGAGCAAGCGTGGCATCCTGA	109
		R: CGCAGCTCGTTGTAGAAGGTGT	
Reference 2	GAPDH	F: ATGAAGCCTGCCCAGATTCC	122
		R: ACCACCTTTTTGGCTCCTCC	

**Table 2:** Statistical results of ovarian transcriptome assembly data.

Summary	Number of Genes	Percentage (%)
Statistics of transcriptome assembly		
Total transcripts	163,186	
Total Unigenes	63,500	
Longest Unigene (bp)	22,653	
Shortest Unigene (bp)	301	
N50 transcript length (bp)	2111	
N50 Unigene length (bp)	1693	
GC_pct		51.83–54.37 (mean = 53.61)
Statistics of transcriptome annotation		
Annotated in NR	24,796	39.04
Annotated in NT	9353	14.72
Annotated in KEGG	10,887	17.14
Annotated in SwissProt	16,807	26.46
Annotated in PFAM	21,555	33.94
Annotated in GO	21,552	33.94
Annotated in KOG/COG	8855	13.94
Annotated in all databases	3305	5.2
Annotated in at least one database	32,440	51.08
Total number of CDS	29,695	46.76
Number of transdecoder-predicted CDS	9859	33.2
Number of CDS searched in databases	19,836	66.8

Note: NR (NCBI non-redundant protein sequences), NT (NCBI nucleotide sequences), KEGG (Kyoto Encyclopedia of Genes and Genomes), SwissProt (a manually annotated and reviewed protein sequence database), PFAM (Protein family), GO (Gene Ontology), KOG/COG (COG: Clusters of Orthologous Groups of proteins; KOG: euKaryotic Ortholog Groups), CDS (coding sequence).

**Table 3:** The top 10 up-regulated and top 10 down-regulated DEGs in the *A. persicus* ovarian transcriptome during the blood-feeding period (OV1 vs. OV0).

GeneID	FPKM (OV0)	FPKM (OV1)	log2FC	padj	NR Description
Cluster-2778.40968	0	7.98	8.0112	1.58 × 10^−10^	serine protease
Cluster-2778.8195	0	2.653	7.5078	4.66 × 10^−9^	--
Cluster-2778.10352	0.073	9.8267	7.1624	9.35 × 10^−9^	serine protease
Cluster-2778.41816	0	8.8467	6.9324	2.55 × 10^−7^	--
Cluster-2778.6648	0	2.61	6.8712	2.67 × 10^−7^	--
Cluster-2778.7580	0.03	3.66	6.8066	7.71 × 10^−8^	serine protease
Cluster-2778.40652	0	2.4067	6.7153	1.29 × 10^−6^	--
Cluster-2778.38220	0	2.33	6.461	3.51 × 10^−6^	--
Cluster-2778.9289	0.22	19.067	6.4352	7.43 × 10^−113^	Na(+)/citrate cotransporter
Cluster-2778.8272	0	1.68	6.3875	4.76 × 10^−6^	--
Cluster-2778.37873	0.403	0	−5.7259	0.0002864	--
Cluster-2778.15372	0.983	0	−5.7556	0.00052655	tcb1
Cluster-2778.38261	2.22	0	−5.9901	9.37 × 10^−5^	--
Cluster-2778.11877	2.247	0	−6.3043	9.11 × 10^−6^	--
Cluster-2778.38351	1.59	0	−6.4119	9.02 × 10^−6^	cubilin
Cluster-2778.17280	2.02	0	−6.5729	1.98 × 10^−6^	MPV17L
Cluster-2778.15450	1.703	0	−6.626	1.45 × 10^−6^	
Cluster-2778.22920	3.443	0	−6.6583	1.61 × 10^−6^	--
Cluster-2778.21097	6.763	0.0567	−6.7136	1.46 × 10^−7^	Allergen
Cluster-13619.0	0.823	0	−6.7309	1.21 × 10^−6^	--

Note: log2FC: log2(FoldChange); a positive value of log2FC suggests gene up-regulation, while a negative value indicates gene down-regulation. The symbol “--” signifies that the gene is not annotated in the NR database.

**Table 4:** The top 10 up-regulated and top 10 down-regulated DEGs in the *A. persicus* ovarian transcriptome during the preoviposition period (OV2 vs. OV1).

GeneID	FPKM (OV1)	FPKM (OV2)	log2FC	padj	NR Description
Cluster-2778.43242	0.07	646.643	13.163	2.44 × 10^−90^	--
Cluster-2778.11888	0.593	1205.44	10.98	0	--
Cluster-2778.42853	0.13	244.063	10.93	1.30 × 10^−120^	--
Cluster-2778.23188	0.043	70.457	10.619	1.97 × 10^−36^	--
Cluster-2778.18139	0.237	266.783	10.146	1.50 × 10^−49^	--
Cluster-2778.9849	0	208.253	9.998	3.62 × 10^−16^	--
Cluster-2778.46089	0.05	43.693	9.9447	1.30 × 10^−126^	vitellogenin
Cluster-2778.31631	1.27	1224.01	9.9275	0	--
Cluster-2778.1351	0	29.927	9.924	4.18 × 10^−16^	monotonin
Cluster-2778.1477	0	65.35	9.6753	1.31 × 10^−15^	Kunitz-type serine protease inhibitors
Cluster-2778.40730	1.21	0	−5.6285	0.00024	--
Cluster-2778.8690	1.837	0	−5.6349	0.000274	--
Cluster-2778.36369	1.627	0	−5.7533	0.000128	--
Cluster-2797.0	3.157	0	−5.7766	0.000137	--
Cluster-2987.0	1.253	0	−5.8111	0.000108	--
Cluster-2778.41221	1.487	0	−5.8152	0.000175	--
Cluster-2778.20830	4.417	0	−5.9664	5.37 × 10^−5^	--
Cluster-2778.40843	1.293	0	−6.1418	2.58 × 10^−5^	--
Cluster-2778.40652	2.407	0	−6.7034	1.59 × 10^−6^	--
Cluster-2778.7580	3.66	0.033	−6.7942	8.86 × 10^−8^	serine protease

Note: log2FC: log2(FoldChange); a positive value of log2FC suggests gene up-regulation, while a negative value indicates gene down-regulation. The symbol “--” signifies that the gene is not annotated in the NR database.

**Table 5:** Hub genes related to ovarian development during the blood-feeding period (OV1 vs. OV0).

Gene ID	Name	Score	FPKM		log2FC	Annotation
			OV0	OV1		
Cluster-2778.209	LRRK1	39	2.2	0.057	−5.0273	leucine-rich repeat serine/threonine-protein kinase 1
Cluster-2778.34485	RPS3A	5	14.78	6.96	−1.0684	40S ribosomal protein S3a
Cluster-2778.38419	H4	5	0.91	2.33	1.3937	Histone 4
Cluster-2778.13663	FabG	4	3.57	10.79	1.6286	3-oxoacyl-[acyl carrier protein] reductase
Cluster-2778.24606	FUM	4	2.34	0.62	−1.8773	fumarase
Cluster-2778.21121	PIK3Cb	4	1.77	0.49	−1.755	phosphatidylinositol 4,5-bisphosphate 3-kinase catalytic subunit beta isoform
Cluster-2778.34617	PDI3	3	5.23	10.557	1.0391	protein disulfide isomerase 3
Cluster-2778.26261	PDI	3	126.82	60.43	−1.0714	protein disulfide isomerase
Cluster-2778.24796	PTEN	3	2.83	9	1.6946	phosphatidylinositol triP phosphatase
Cluster-2778.29372	HSP70	3	106.83	218.94	1.0625	heat shock protein 70

Note: “Score indicates the degree of nodes in the PPI network; log2FC: log2FoldChange.

**Table 6:** Hub genes related to ovarian development during the preoviposition period (OV2 vs. OV1).

Gene ID	Name	Score	FPKM		log2FC	Annotation
			OV1	OV2		
Cluster-2778.23724	APRT	14	13.57	34.25	1.3188	adenine phosphoribosyltransferase
Cluster-2778.29659	RPL13A	14	437.09	876.26	1.0599	ribosomal protein L13A
Cluster-2778.34298	TnI	13	3.31	15.87	2.2441	troponin I protein
Cluster-2778.22975	PRM	12	3.93	27.59	2.7926	paramyosin
Cluster-2778.30915	MyHC	12	2.64	16.04	2.5853	myosin heavy chain
Cluster-2778.38419	H3	12	2.3	1.1	−1.1013	Histone 3
Cluster-2778.17397	HEX (HEXB)	11	1.38	8.19	2.5592	beta-hexosaminidase subunit beta
Cluster-2778.32843	HEX (HEXA)	11	6.13	29.88	2.2908	beta-hexosaminidase subunit alpha
Cluster-2778.33259	ENH	10	2.53	6.92	1.4376	adaptor protein enigma
Cluster-2778.26644	HSDL2	10	1.55	3.37	1.1192	hydroxysteroid dehydrogenase protein 2

Note: “Score” indicates the degree of nodes in the PPI network; log2FC: log2FoldChange.

## Data Availability

All data obtained in this study was deposited at the National Center for Biotechnology Information (NCBI) with the accession number PRJNA1285112.

## Supplementary Materials

The following supporting information can be downloaded at: https://www.mdpi.com/article/10.3390/genes16091107/s1, Figure S1 and Tables S1: Assessing the completeness of *Argas persicus* ovary transcriptome assembly using BUSCO. Tables S2–S3: Hub genes related to ovarian development during the feeding period (OV1 vs OV0) and the preoviposition period (OV2 vs OV1), respectively.

## Abbreviations

The following abbreviations are used in this manuscript:

*A. persicus*	*Argas persicus*
DEGs	differentially expressed genes
PPI	protein–protein interaction
LD	linear dichroism
HSP70	heatshockprotein70
PDI	protein disulfide isomerase
PDI3	protein disulfide isomerase 3
PRM	paramyosin
TnI	troponin I protein
HEX	beta-hexosaminidase
Vg	vitellogenin
Vn	vitellin
*H. longicornis*	*Haemaphysalis longicornis*
