# Supplementary material for: De Novo Transcriptome Sequencing and Profiling of Ovarian Development of Argas persicus Along the Trophogonic Cycle
Source: Genes (Basel). 2025 Sep 19;16(9):1107. doi: 10.3390/genes16091107 (PMC12469708; doi:10.3390/genes16091107)
Supplement: Supplementary file 1 [file genes-16-01107-s001.zip › genes-3784535-supplementary.pdf]

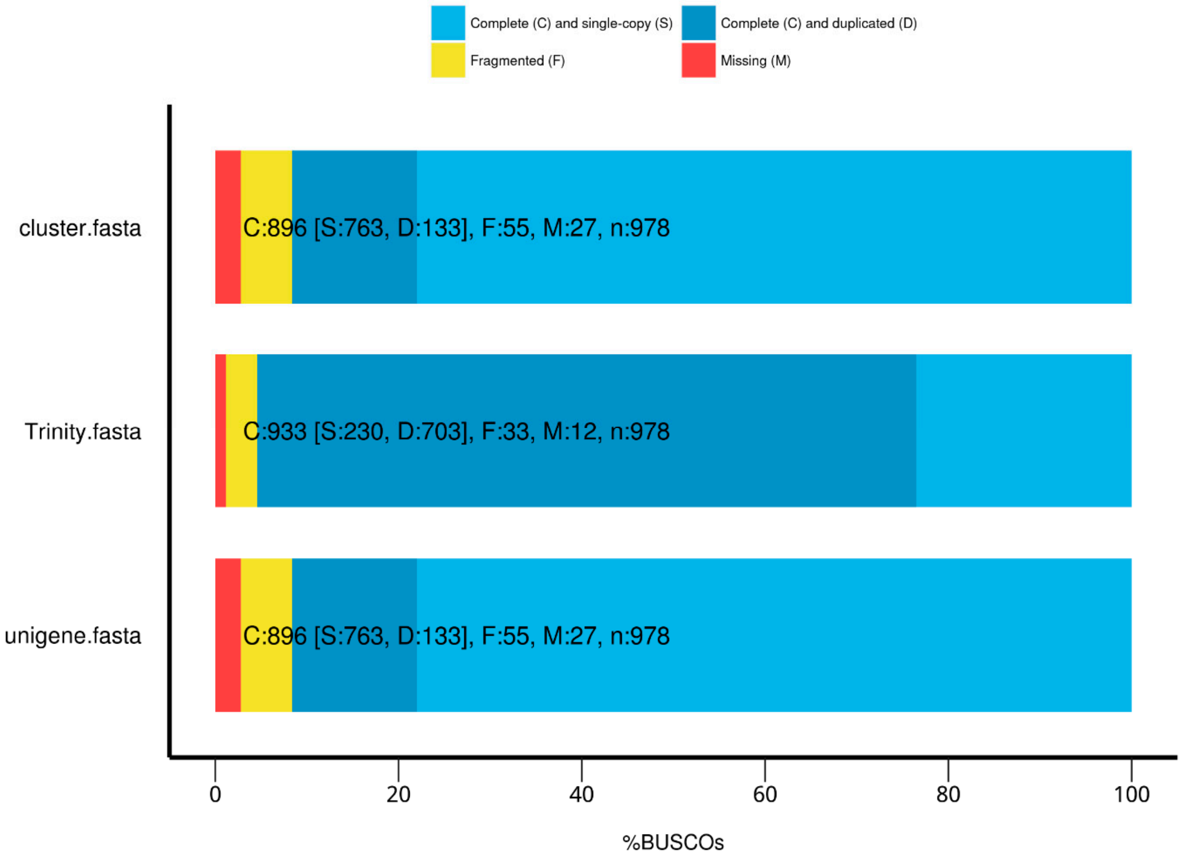

Figure S1. Assessing the completeness of *Argas persicus* ovary transcriptome assembly using BUSCO (https://busco.ezlab.org/). Trinity.fasta refers to the sequence assembled by the Trinity software. Cluster.fasta is generated by clustering and removing redundancies from Trinity.fasta using the Corset tool. The longest transcript for each gene in cluster.fasta is selected as the unigene for that gene and is subsequently stored in unigene.fasta.

Table S1. Assessing the completeness of Argas persicus ovary transcriptome assembly using BUSCO.

type	Complete(single)	Complete(duplicated)	Complete BUSCOs (C)	Fragmented	Missing
cluster	763(78.0%)	133(13.6%)	896（91.6%）	55(5.6%)	27(2.8%)
unigene	763(78.0%)	133(13.6%)	896（91.7%）	55(5.6%)	27(2.8%)
Trinity	230(23.5%)	703(71.9%)	966（95.4%）	33(3.4%)	12(1.2%)

Table S2. Hub genes related to ovarian development during the feeding period (OV1 vs OV0)

Gene ID	Name	Best Match					Match to *Ixodes scapularis* (ID: 6945)			
		Entry	tick species	Identity (%)	Score	E-Value	Entry	Identity (%)	Score	E-Value
Cluster-2778.209	LRRK1	B7PZ63	*I. scapularis*	39.4	173	6.70E-14	B7QGJ2	81.4	73.6	7.90E-14
Cluster-2778.34485	RPS3A	A0A9J6F420	*R. microplus*	82.1	175	2.50E-17	B7PSQ6	76.2	69.7	4.50E-13
Cluster-2778.38419	H4	A0A6M2CV35	*R. microplus*	100	679	5.10E-91	Q4PM69	99.0	199.9	4.50E-52
Cluster-2778.13663	FabG	A0A1Z5KU47	*O. moubata*	71.8	367	5.80E-42	B7Q7G3	71.0	141.7	1.30E-34
Cluster-2778.24606	FUM	B7PXR2	*I. scapularis*	87.5	280	4.70E-33	B7PXR2	87.5	102.4	6.00E-23
Cluster-2778.21121	PIK3Cb	A0A2R5LDE5	*O. turicata*	92.1	874	2.00E-112	B7Q6K8	87.0	324.7	1.90E-89
Cluster-2778.34617	PDI3	B7P3N0	*I. scapularis*	55.9	1664	0	B7P3N0	55.9	650.2	6.30E-187
Cluster-2778.26261	PDI	A0A293MN96	*O. erraticus*	86.8	635	7.30E-81	B7P4U1	75.7	218	1.90E-57
Cluster-2778.24796	PTEN	A0A9D4Q7X0	*R. sanguineus*	58.5	1037	3.00E-137	B7Q941	31.6	168.7	5.30E-42
Cluster-2778.29372	HSP70	A0A2R5LEC1	*O. turicata*	89	2913	0.00E+00	B7QL71	84.8	1036.6	3.50E-303

Note:

(1) "Best match" refers to the tick protein with the highest sequence similarity to the gene-encoded CDS sequence in the UniprotKB database; "Entry" and "tick species" refers to the tick protein information of "Best match".

(2) " Match to Ixodes scapularis (ID: 6945) " refers to the results of homology comparisons between the *Argas persicus* protein and its homologous proteins found in the *Ixodes scapularis* genome (NCBI taxonomy ID: 6945); "Entry" refers to the specific protein ID associated with the *Ixodes scapularis* genome.

Table S3. Hub genes related to ovarian development during the preoviposition period (OV2 vs OV1)

Gene ID	Name	Best Match					Match to *Ixodes scapularis* (ID: 6945)			
		Entry	tick species	Identity (%)	Score	E-Value	Entry	Identity (%)	Score	E-Value
Cluster-2778.23724	APRT	A0A293N4F0	*O. erraticus*	68.9	495	2.70E-63	B7Q9G6	64.7	174	3.50E-44
Cluster-2778.29659	RPL13A	A0A1Z5LHG9	*O. moubata*	89.2	817	9.20E-112	B7PR90	87.3	313	6.90E-86
Cluster-2778.34298	TnI	Q969A1	*H. longicornis*	84.2	804	1.60E-109	B7QKT0	80	186	1.50E-47
Cluster-2778.22975	PRM	A0A1Z5KV26	*O. moubata*	92.1	4028	0	B7P5D8	85.6	289	7.60E-78
Cluster-2778.30915	MyHC	A0A131Z6U4	*R. appendiculatus*	92.6	9135	0	B7P1Q2	95.6	117	6.50E-26
Cluster-2778.38419	H3	A0A1Z5L7F9	*O. moubata*	100	675	9.80E-91	B7P7T4	97.1	249	1.40E-66
Cluster-2778.17397	HEXB	A0A293N5I8	*O. erraticus*	83.4	2521	0	B7PY88	80.9	286	3.20E-77
Cluster-2778.32843	HEXA	A0A293MR42	*O. erraticus*	65.6	996	5.10E-131	B7PE22	66.2	200	1.00E-51
Cluster-2778.33259	ENH	V5HPX6	I. ricinus	60.7	2499	0	B7QCQ7	40.2	488	5.20E-138
Cluster-2778.26644	HSDL2	A0A2R5L577	O. turicata	86.6	539	4.80E-71	B7PKM5	55.6	113	1.20E-25

Note:

(1) "*Best* match" refers to the tick protein with the highest sequence similarity to the gene-encoded CDS sequence in the UniprotKB database; "Entry" and “tick species” refers to the tick protein information of "Best match".

(2) " Match to Ixodes scapularis (ID: 6945) " refers to the results of homology comparisons between the *Argas persicus* protein and its homologous proteins found in the *Ixodes scapularis* genome (NCBI taxonomy ID: 6945); "Entry" refers to the specific protein ID associated with the *Ixodes scapularis* genome.
